# ABO-incompatible kidney transplantation with high isoagglutinin titres: practice pattern and problem solving: a German multi-centre survey

**DOI:** 10.3389/fimmu.2026.1831754

**Published:** 2026-06-05

**Authors:** Stefan Reuter, Christian Jungck, Ana Harth, Jeannine Wegner, Wolfgang Arns, Katrin Ivens, Anja Mühlfeld

**Affiliations:** 1 Department of Medicine D, Transplant Nephrology, University Clinics Münster, Münster, Germany; 2 Medizinische Klinik I (Klinik für Nephrologie, Transplantationsmedizin und internistische Intensivmedizin), Kliniken der Stadt Köln, Cologne, Germany; 3 Medical Center Cologne-Merheim, University Witten/Herdecke, Cologne, Germany; 4 German Living Donation Register, University of Münster, Münster, Germany; 5 Department of Nephrology, Faculty of Medicine, University Hospital, Heinrich-Heine-University, Düsseldorf, Germany; 6 Division of Nephrology and Immunology, University Hospital Rheinisch-Westfälische Technische Hochschule Aachen (RWTH) Aachen, Aachen, Germany

**Keywords:** ABO incompatible transplantation, desensitisation, high isoagglutinin titres, kidney transplantation, renal transplant

## Abstract

**Introduction:**

ABO-incompatible kidney transplantation (ABOi-KT) expands the donor pool, yet baseline anti-ABO isoagglutinin titres ≥ 1:512 remain challenging and practice is highly variable. We surveyed the German transplant centres to capture current management of very high-titre ABOi candidates and centre responses to insufficient titre decline.

**Methods:**

Thirty-six centres were invited to complete an anonymised 19-item web-based questionnaire (January–March 2025) on (i) centre activity, (ii) isoagglutinin testing and isoagglutinin titre limits (iii) desensitisation protocol, (iv) ABO abort criteria and (v) escalation & re-attempt strategies. Data are reported descriptively.

**Results:**

Of 36 eligible programmes, 27 (75%) responded. Thirteen (48%) accept any isoagglutinin titre; the rest cap at 1:512 (15%), 1:1024 (26%) or 1:2048 (11%). Centres without a cap reported greater 5-year ABOi experience (median 20 vs 9 cases). IgG isoagglutinin titres are monitored in 26/27 centres, IgM in 18/27. All centres use rituximab, usually 4 weeks pre-transplant. Antigen-specific immunoadsorption (IA) is the principal antibody-removal method in 88%, while 54% also employ plasma exchange (PLEX). In cases of insufficient titre decline, different strategies are employed. Desensitisation is halted mainly for refractory isoagglutinin titres or serious complications; 67% of programmes would re-attempt after 3–6 months, typically with intensified apheresis and/or additional B-cell depletion.

**Discussion:**

German centres display marked protocol heterogeneity, but all accept very high titres, relying on rituximab plus IA. Greater experience correlates with abandonment of fixed cut-offs. Harmonised isoagglutinin titre assays, evidence-based futility criteria and multicentre registries are needed to optimise high-titre ABOi-KT.

## Graphical Abstract

**Figure:**
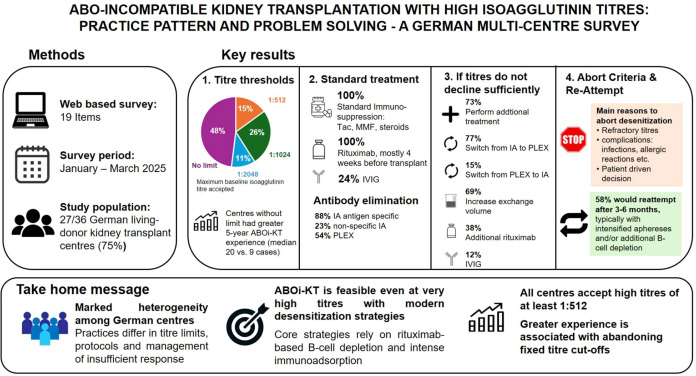

## Introduction

The elimination of blood-group barriers has been identified as a proven strategy for the expansion of the living-donor kidney pool. Following the establishment of the first routine programmes in Japan, ABO-incompatible kidney transplantation (ABOi-KT) has become mainstream in Europe and North America, accounting for 10–30% of living donations in high-volume centres. A 2018 meta-analysis of >3,000 recipients confirmed that modern ABOi-KT achieves excellent 1year-graft and patient survival rates (96% vs. 98% in ABO compatible KT), albeit with a moderately higher incidence of antibody-mediated rejection (AMR) and infections (1). These findings are supported by a large Collaborative Transplant Study analysis, which demonstrated comparable 3-year death-censored graft survival and overall patient survival relative to ABO-compatible controls, albeit with increased early infectious mortality (2).

Long term studies confirmed excellent patient and graft survival that approaches that of ABO-compatible transplantation (3, 4). These advances can be attributed to two major developments: the first is the use of anti-CD20 B-cell depletion with rituximab, which obviated the need for splenectomy (5); the second is the development of highly efficient extracorporeal antibody-removal techniques, such as antigen-specific immunoadsorption (IA) (4). Most centres administer a single 375 mg/m² dose of rituximab around four weeks pre-transplant to deplete CD20^+^ B cells (5). Anti-A/B antibodies are then removed using IA and/or plasma exchange (PLEX), with or without intravenous immunoglobulins (IVIGs). In principle, there are two types of IA column: specific and unspecific. Both enable safe isoagglutinin titre reduction, but non-specific columns may reduce cost at the expense of time efficiency and safety as they deplete also protective antibodies (6).

### The challenge of very high titres

Despite the overall success of ABOi-KT, recipients who enter desensitisation with very high baseline anti-A/B isoagglutinin titres (≥ 1:512) remain clinically challenging (7). Recent evidence confirms that ABO-incompatible KT remains feasible even at very high baseline isoagglutinin titres when modern desensitisation and vigilant aftercare are applied, with favourable graft and patient survival in experienced programmes (8). However, elevated titres prolong desensitisation, increase resource utilisation and bleeding risk, and correlate with steep post-operative antibody rebound leading to higher AMR rates and shorter graft survival (7–9). No international guideline currently defines an upper titre limit or futility benchmark. Programmes worldwide employ ceiling values ranging from 1:256 to > 1:2048, or none at all. The interpretation of these results is further complicated by the absence of a standard laboratory assay for isoagglutinin measurement. Clinicians must therefore balance the achievement of a safe peri-operative isoagglutinin titre (commonly ≤ 1:8–1:16 IgG) against the toxicity and cost of aggressive desensitisation.

### Insufficient decline of isoagglutinin titres

In clinical practice, titres often decrease by approximately one titre step per treatment. However, it should be noted that in some cases, the titres may not fall as predicted or exhibit marked rebounds. The clinician must decide whether to continue with the treatment, to add extracorporeal treatment (if so, how much)?, to implement other additional strategies, or to cancel the planned transplantation procedure. As we found no published guidance or strategies for this situation, we therefore conducted a national survey to ascertain possible remedies.

### The German situation, heterogeneous practice and the German gap

Organ donation rates in Germany remain significantly lower than in many other Western countries, resulting in prolonged waiting times for deceased-donor kidney transplantation. In this context, living-donor kidney transplantation represents an important strategy to improve access, accounting for approximately 25–30% of all kidney transplants performed annually.

In contrast to several other countries, however, structured kidney paired exchange (KPE) programmes are not yet widely implemented in Germany. In countries such as the United States, the United Kingdom, the Netherlands and South Korea, KPE has become an established strategy to overcome immunological incompatibilities, particularly ABO incompatibility, thereby reducing the need for high-risk desensitisation protocols (10, 11). In these settings, patients with very high isoagglutinin titres can often be redirected into exchange programmes rather than undergoing intensive ABO-incompatible transplantation. In Germany, cross-over living donation is currently limited and is only expected to be implemented more broadly with the anticipated amendment of the German Transplantation Act (expected in 2026). As a consequence, transplant centres may face a structural gap: in the absence of widely available exchange options, ABO-incompatible living-donor transplantation remains one of the few viable strategies to avoid prolonged dialysis in otherwise suitable donor–recipient pairs. This situation may contribute to a greater willingness of German centres, compared with their international counterparts, to accept very high isoagglutinin titres and to pursue more intensive desensitisation strategies. At the same time, this structural context may also explain the considerable heterogeneity observed in clinical practice, as centres balance immunological risk, procedural burden and the lack of alternative pathways. However, national guidance for ABO incompatibility is lacking. Informal reports suggest that there is considerable variability in the approach adopted by different centres. Some centres proceed regardless of starting isoagglutinin titre whereas others decline candidates with isoagglutinin titres ≥1:512. There is an absence of a German registry that systematically captures such protocols or outcomes, which hinders the refinement of practice on an evidence-based basis.

### Study objectives

To delineate real-world management of ABOi-KT in recipients with baseline isoagglutinin titres ≥1:512, we conducted an anonymised nationwide survey of German living-donor transplant centres. The objectives of the present study were to (i) catalogue centre-specific isoagglutinin titre thresholds and desensitisation protocols, (ii) compare strategies between programmes that do or do not impose an upper limit, (iii) identify strategies dealing with failure to achieve reduction of isoagglutinin levels to target titre with the planned protocol, and (iv) provide options for clinicians in individual situations. The mapping of this heterogeneity should inform future guideline development and improve outcomes for the subgroup of ABO-incompatible transplantation at highest risk.

## Materials and methods

### Study design and study group

We conducted a national, cross-sectional, web-based survey of German kidney-transplant programmes. The survey was created after expert discussion within the Working Group of NRW Transplant Physicians (Arbeitsgemeinschaft der Nierentransplantationszentren Nordrhein-Westfalens). As of 31 December 2024, 39 German hospitals were performing adult kidney transplants. Three of these hospitals do not offer living-donor procedures and were therefore excluded. The remaining 36 living-donor centres, which cover all seven German transplant regions, formed the sampling frame.

### Questionnaire

A survey was created in SurveyMonkey^®^ after reviewing key ABOi-KT desensitisation literature. The final survey comprised 19 items in five domains (**Table 1**): centre profile and activity (1), isoagglutinin testing and titre limits (3), desensitisation protocols (8), ABOi-KT abort/futility criteria (1) and escalation and re-attempt strategies (6). A complete set of questions is provided in **Supplementary Table 1**. On 8 January 2025, personalised email invitations containing a unique link were sent to the medical director or the lead transplant nephrologist/surgeon at each centre. Two automated reminders were sent at 14-day intervals, and the survey closed on 31 March 2025. IP address checks prevented duplicate submissions and no incentives were offered. The survey data were then exported to Excel (Microsoft, Redmond, USA) for analysis. Open-text responses were reviewed by three reviewers, and any disagreements were resolved by consensus.

**Table 1:** Survey questions.

Domain	Survey questions (19 total)
1 Centre profile & activity (1 Q)	Number of ABOi transplants in last 5 years
2 Isoagglutinin testing & titre limits (3 Q)	• Assay platform used (tube, gel, flow) • Isoagglutinin classes measured (IgG/IgM) • Maximum acceptable pre-transplant IgG titre
3 Desensitisation protocol (8 Q)	• Special pre-treatment for high-titre candidates (e.g., intensified/extra rituximab) • Start of oral triple immunosuppression before transplant • Timing of rituximab administration • Routine IVIG use/dosing policy • Primary extracorporeal modality (antigen-specific IA vs. non-specific IA vs. PLEX) • Processed plasma volume per session (IA with antigen-specific columns) • Processed plasma volume per session (IA with non-specific columns) • Processed plasma volume per session (PLEX)
4 Abort/futility criteria (1 Q)	• Predefined Abort/futility criteria (refractory titres, complications; multiple choice+open questions)
5 Escalation & re-attempt strategies (6 Q)	• Escalation strategy when titres plateau (e.g., switch IA↔PLEX, increase volume; adjuncts) • Maximum number of extracorporeal treatments performed before transplant at your centre (historical) • Willingness to re-desensitise after failure • Minimum waiting interval before re-attempt • Conditions under which a re-attempt would be undertaken • Additional measures planned for a re-attempt

### Statistical analysis

Continuous variables are reported as median, inter-quartile range (IQR) and overall range where appropriate. Categorical variables as counts (%), as the number of valid responses varied across questions. Results are presented as fractions and percentage to indicate the respective sample size. No formal hypothesis tests were performed, and no p-values are reported; results are descriptive. It was determined that a formal sample-size calculation was not required, given that the survey targeted the entire national universe of eligible centres (finite-population design).

### Ethics statement

This survey gathered only anonymised, centre-level data from healthcare professionals; no patient or personal information was obtained. Participation was voluntary, and survey completion implied consent. Under German regulations, such activity is exempt from full ethics review.

## Results

### Centre participation and characteristics

Of the 36 German living-donor kidney-transplant programmes that were contacted, 27 (75%) completed the survey. Three centres chose to remain anonymous, one of those stopped completion in the middle of the survey for unknown reasons. Completed answers were still analysed, each centre was asked to report the number of ABO-incompatible kidney transplants performed within the previous five years. Twenty-three centres provided data, comprising a total of 411 ABOi transplants. The median institutional ABOi-KT case-load over the previous five years was 16 transplants (interquartile range [IQR] 8-23.5, total range 3-50), but volumes differed between centres that impose a titre ceiling (median 9) and those that do not (median 20).

### Maximum acceptable baseline isoagglutinin titre

**Figure 1:**
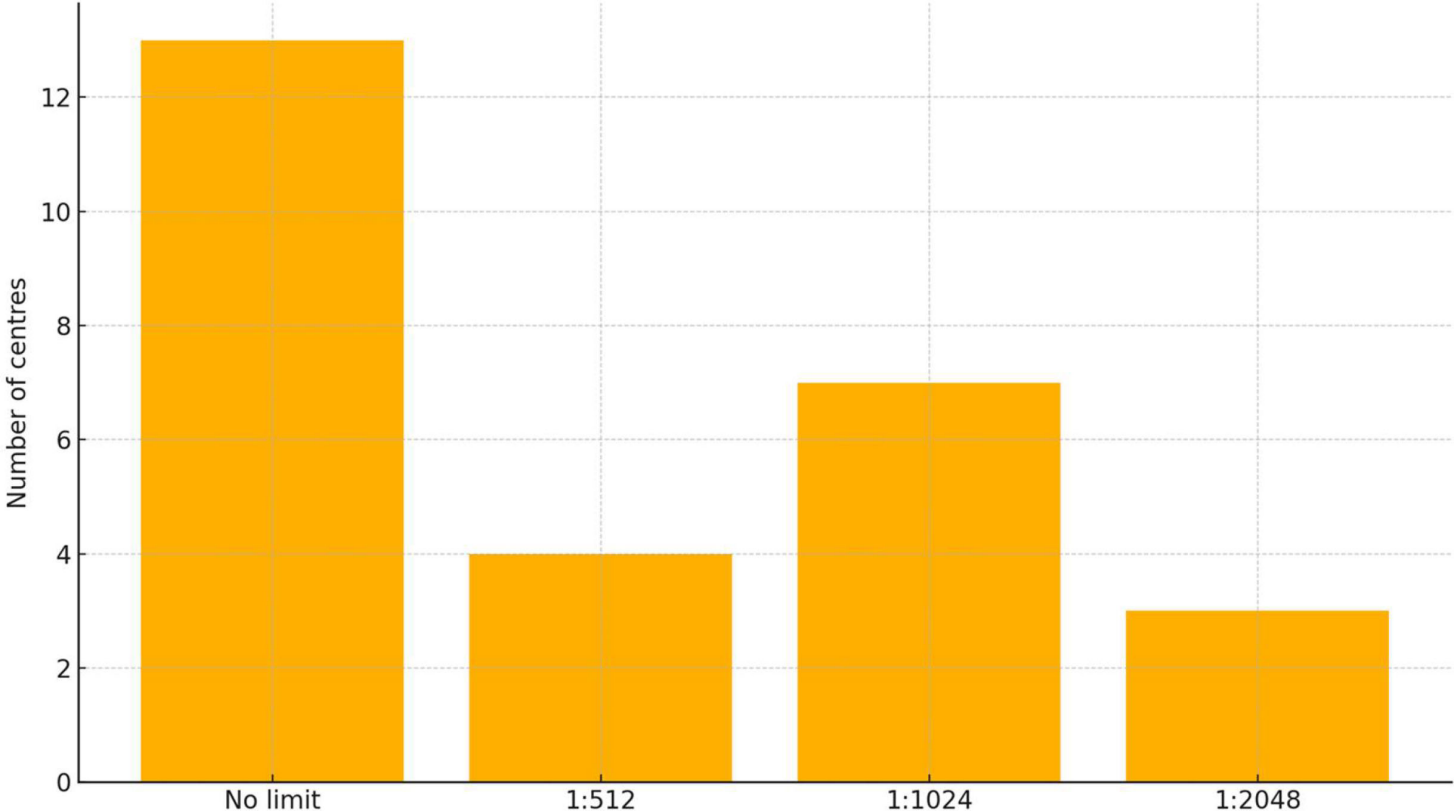
Maximum baseline isoagglutinin titre accepted. Distribution of centre-reported maximum acceptable baseline anti-A/B titres (n = 27).

All 27 centres accepted isoagglutinin titres of at least 1:512. Almost half the programmes (13/27, 48%) reported no upper isoagglutinin titre limit for proceeding to transplantation. Among the remaining 14 centres, limits clustered at 1:512 (4 centres), 1:1024 (7) and >1:2048 (3) (**Figure 1**). Centres without a fixed upper isoagglutinin titre limit reported a higher number of ABOi-KT over the past five years compared with centres applying predefined titre thresholds (median 20 vs. 9 cases). This suggests a potential association between centre experience and the willingness to accept higher baseline titres. However, given the descriptive nature of the survey, the limited sample size, and missing data for some variables, no formal statistical analysis was performed.

### Isoagglutinin-testing practices

IgG isoagglutinin titres were measured in 26/27 centres, while IgM quantification was performed in 18/27 (67%) centres. The predominant method of analysis is tube or gel agglutination, with only one centre employing a flow-cytometric assay.

### Desensitisation protocols for high isoagglutinin titres

Rituximab is used by all centres and is most often administered 4 weeks prior to transplant (64%). However, details of rituximab application showed notable variability. The timing of administration ranged from 5 weeks to 1 day prior to transplantation, with the majority of centres applying rituximab approximately 2–4 weeks before the planned procedure. While a single-dose strategy was standard in most centres, additional dosing strategies were reported in specific high-titre scenarios or during re-attempts after failed desensitisation.

In the high-titre population, 20/26 centres (77%) use only their standard, single dose of Rituximab (375 mg/m² body surface area). One centre administers a dose of 500 mg, 3 centres routinely add a second dose, one adds a second dose only if ABO isoagglutinin titres do not decrease sufficiently after 2 or 3 IA treatments, one centre adds 75 mg of thymoglobuline, and one starts rituximab earlier.

**Figure 2:**
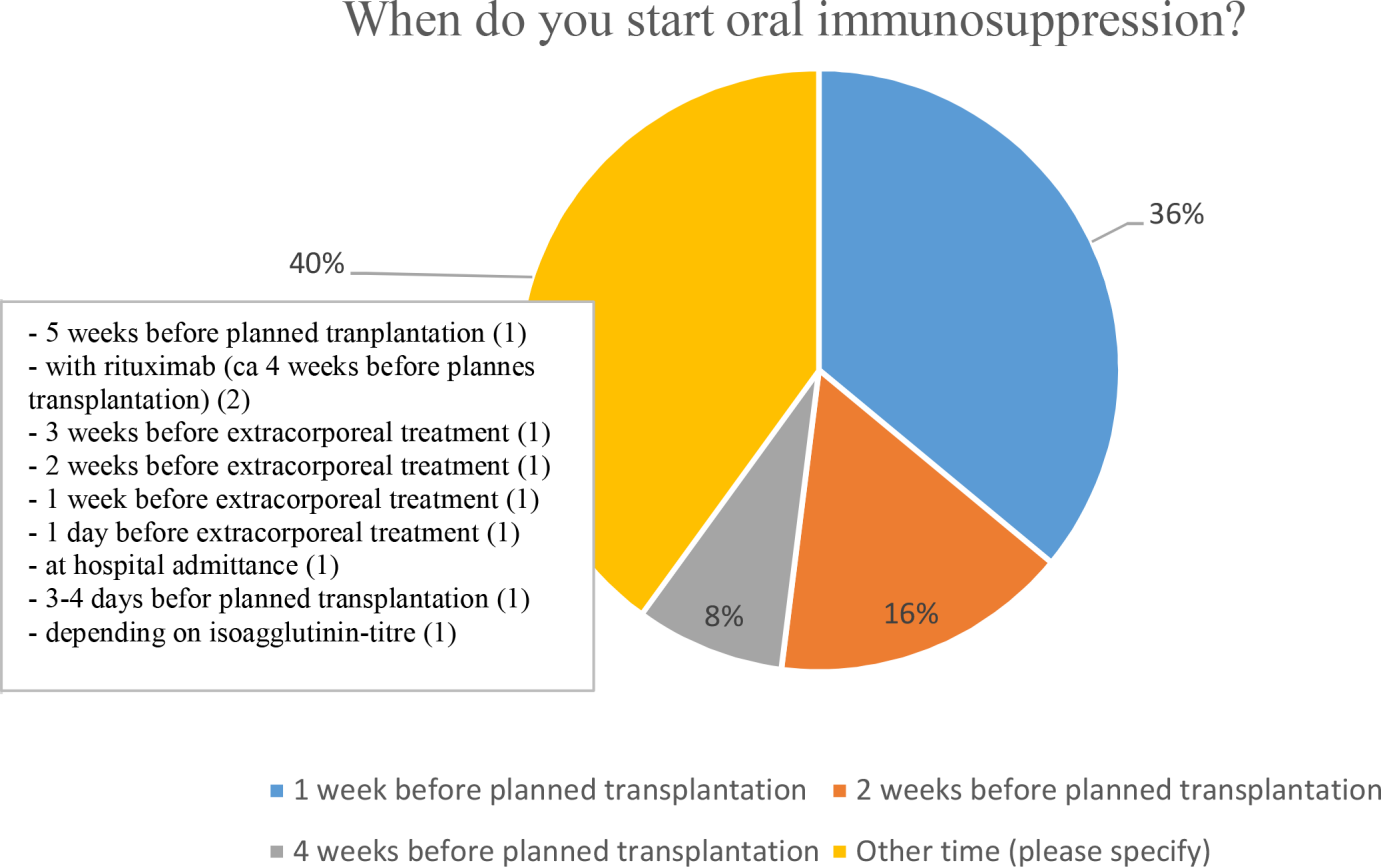
Start of oral immunosuppressive therapy.

Oral immunosuppression is initiated between 5 weeks to 4 days prior to transplant. (**Figure 2**, **Table 2**). One centre differentiates between low (start with IA) and high (start with RTX 4 weeks prior to transplant) isoagglutinin titres.

**Table 2:** Desensitisation protocol details.

Parameter	Most common response
Rituximab	Single 375 mg/m² dose, most commonly ~4 weeks pre-transplant; 26/26 centres use rituximab, one of them conditionally (titres >=1:16)
Start of oral Immunosuppression before ABOi-KT	1 week (36%), 2 weeks (16%), 4 weeks (8%); other (40%)
IVIG use	6/25 (24%), 2/25 only in high titres
Primary extracorporeal modality	Antigen-specific IA 23/26 (88.5%); non-specific IA ~23%; PLEX in 14/26 (53.8%)
Median processed plasma volume (PV) per session	“Standard”: PLEX: 1.4 PV, Antigen-specific IA 3 PV, Non-specific IA 2.5 PV “Increased”: PLEX: 2 PV, Antigen-specific IA 4 PV, Non-specific IA 3 PV
Escalation when titres plateau	Most centres switch modality (IA↔PLEX), perform additional treatments and/or increase processed volume; some add additional IVIG or rituximab

IVIG treatment is given regardless of titre by 6/25 centres (24%) and only in high-titre patients by another 2 (8%). 68% of centres do not use IVIG in this setting.

Antigen-specific immunoadsorption was the preferred apheresis method, used by 23 out of 26 centres (88%). Six centres (23%) also employed non-specific columns, and more than half of the programmes (14 out of 26, or 54%) incorporated plasma exchange (PLEX). In cases with high isoagglutinin titres 50% (7/14) of centres reported increasing the treated PV when using ABO-specific and 50% (4/8) when using AB-unspecific columns, only 19% (3/16) did so with plasma exchange. There was a high rate of missing (22%-33%) and a few implausible answers for this item. “Standard” ABO-specific therapy treated a median of 3 PV (IQR 2.5-3; Range 1-5), “standard” ABO-unspecific therapy a median of 2.5 PV (IQR 2.3-4; Range 1.5-5) and “standard” PLEX a median of 1.4 PV (IQR 1-2.1; Range 1-3). “Increased volume” ABO-specific therapy treated a median of 4 PV (IQR 3-4; Range 1.5-6), ABO-unspecific therapy a median of 3 PV (IQR 2,75-3,75; Range 2-6) and PLEX a median of 2 PV (IQR 2-2.5; Range 2-3). Some centres only reported fixed absolute volumes for specific columns (14 and 12 litres), unspecific columns (12 litres) and plasmapheresis (2.5, 2-2.5, 2–3 and up to 4 litres) regardless of isoagglutinin titre.

All centres were asked to report the highest number of extracorporeal treatments used in an individual patient that resulted in a successful transplant. Across centres that provided data, the maximum number of pre-transplant extracorporeal sessions in a single recipient was 36. The median of treatment maxima was 17 (interquartile range (IQR) 10–24, range 5–36). This highlights a substantial variability in procedural intensity between centres The maximum number of extracorporeal treatments administered to an individual patient that did not lead to a successful transplant was 30.

### Management of insufficient isoagglutinin titre decline or rebound

**Figure 3:**
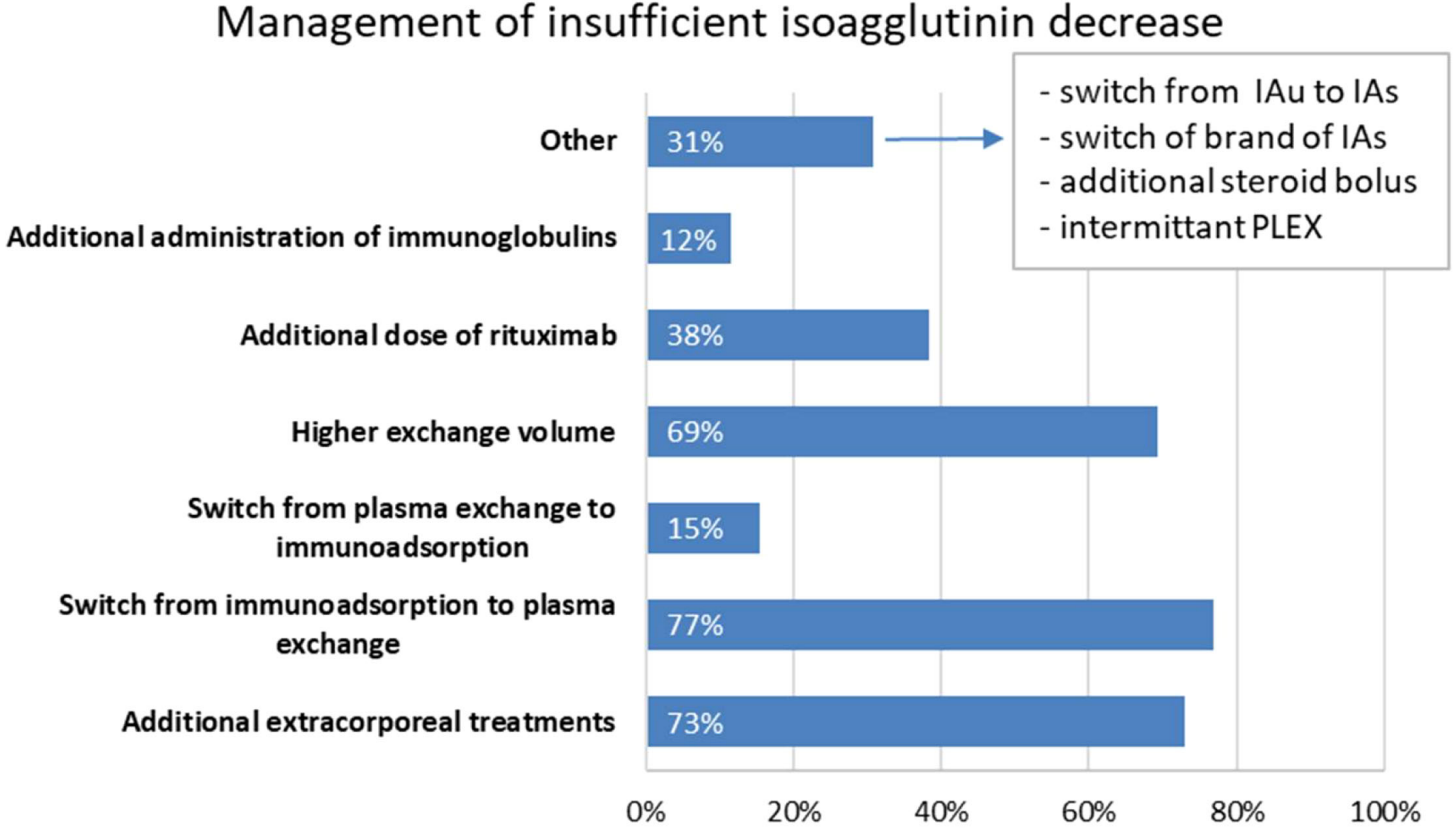
Management of insufficient isoagglutinin titre decrease.

In cases where ABO isoagglutinin titres do not decline sufficiently, 96% (25/26) of centres reported escalation of desensitisation therapy. The most frequently applied strategies were switching from IA to PLEX 77% (20/26), performing additional extracorporeal treatments 73% (19/26) and increasing the exchange volume (69%, 18/26), whereas switching from PLEX to IA was less common (15%, 4/26). Pharmacological escalation was reported less frequently, with 38% (10/26) of centres administering an additional dose of rituximab and 12% (3/26) IVIG. Additional measures were reported only sporadically (each by a single centre) and included switching the brand of specific adsorbers, changing from non-specific to antigen-specific adsorbers, and administration of a high-dose steroid bolus (500 mg). These strategies were often applied in combination rather than in isolation, reflecting both a consistent core escalation pattern and considerable variability in centre-specific adaptations (**Figure 3**).

### Abort/futility criteria

Desensitisation was most commonly discontinued due to insufficient titre reduction or the occurrence of clinically relevant complications. Additional reasons included patient preference. Only 2/24 centres explicitly state to have never stopped desensitisation before. 16/20 (80%) centres reported termination of preparatory procedures owing to a recurrent antibody rebound exceeding the centre-specific isoagglutinin titre threshold. Other triggers were complications of therapy: infections (4), allergic reactions (3), overall clinical condition (2), thrombocytopenia (1) and hypocalcaemia (1) as well as other medical problems (1) and posterior reversible encephalopathy syndrome (1). Noteworthy, 3/23 (13%) centres reported patient-driven discontinuation of therapy. Interestingly, 4 centres (including two large volume centres) reported fixed numeric caps on extracorporeal therapy numbers (7–12 sessions), while 2 reported somewhat flexible caps of about 10 or about 20 extracorporeal treatments.

### Re-attempt policies

More than half of centres reported a willingness to reattempt desensitisation after an interval (typically 3–6 months), usually with protocol intensification. In detail, a second desensitisation attempt would be considered by 67% (16/24) of programmes. 20% (3/15) of centres would restart after three months, 47% (7/15) after six months and the rest on a case-by-case basis (range: 4 weeks to 6 months). In an open-ended question regarding the conditions for a renewed attempt, 5 out of 15 centres cited patient preference, while 3 centres would consider a second attempt only if the previous discontinuation had been unrelated to isoagglutinin titre rebound. Additional measures with a re-attempt would be employed by 8/16 (50%) of centres, most often repeat rituximab (6/16), but also a change of column or an increase in treated plasma-volume or use of obinutuzumab (1 centre).

Overall, the survey demonstrates a high degree of heterogeneity across all domains, including titre thresholds, desensitisation timing, extracorporeal strategies, and escalation pathways.

## Discussion

With responses from 27 of 36 eligible German living-donor kidney transplant programmes (75%), this survey provides a representative snapshot of how ABOi-KT is managed in candidates with very high baseline isoagglutinin titres in Germany. To our knowledge, this is the first national survey focusing specifically on centre-defined upper titre policies in “high-titre” ABOi-KT and practical escalation strategies when titres decline insufficiently.

Three key findings emerge. First, acceptance of baseline titres ≥1:512 appears to be routine: all responding centres reported performing ABOi-KT at least up to 1:512, and almost half reported no fixed upper baseline titre limit. Second, while core elements of desensitisation are widely shared—rituximab-based B-cell depletion combined with intensive extracorporeal antibody removal (predominantly antigen-specific IA, often complemented by plasma exchange)—the pathways used to achieve “transplantability” vary considerably across centres. Third, stopping rules, abort criteria, and re-attempt strategies are heterogeneous and frequently driven by complications, patient preference, and logistics in addition to immunological response. A plausible contextual driver is the German deceased-donor waiting-time environment. Under ETKAS, prolonged waiting times (about double or more of the waiting times in other ET countries (12),) increase the clinical pressure to realise transplantation when a willing living donor is available. In such a setting, centres and recipients may accept increased procedural burden and immunological risk to avoid extended dialysis exposure, particularly since ABO-incompatible living-donor kidney transplantation has been associated with a survival advantage compared with remaining on the waiting list (7–9, 13).

Even though high titre transplants seem to be standard, the survey data shows wide heterogeneity in the ways to achieve transplantability (for example the different starting dates of rituximab and oral immunosuppression, or the use of different extracorporeal treatment modalities). This heterogeneity likely reflects both a limited comparative evidence base and differences in local infrastructure and reimbursement that shape what is feasible. As in ABOi-KT in general, to our knowledge there exist no RCTs comparing different induction protocols, most data consist of (long term) cohort studies or inter-centre comparisons before and after changes in treatment protocol.

Of the 27 programmes that responded, the core desensitisation strategy was rituximab-based B-cell depletion followed by intense apheresis, most commonly antigen-specific IA. This aligns with the evolution of ABOi protocols away from splenectomy towards anti-CD20-based regimens combined with antigen-specific immunoadsorption (4, 5). A majority of centres reported protocol intensification in high-titre candidates or when titres did not fall as expected, including additional plasma exchange, switching between immunoadsorption and plasma exchange, changing adsorber types, and steroid boluses. Repeat rituximab dosing was reported in selected scenarios (pre-specified in high-titre protocols, triggered by insufficient decline, or planned for re-attempts). However, these “second-dose” practices should be considered hypothesis-generating rather than evidence-based at present, particularly given plausible infection and haematologic toxicity trade-offs with higher cumulative B-cell depletion (4, 14).

Substantial session-specific processed volumes were observed: a median of 1.4 PV per PLEX session in the “standard” and two PV in the “increased” group and up to a median of four PV per ABO-specific “increased” IA session. Centres reported very high maximum numbers of extracorporeal sessions in individual cases (up to 36 pre-transplant sessions in a patient who proceeded to transplant, and up to 30 sessions in a patient who ultimately did not), illustrating how far programmes may go to render very high-titre candidates transplantable. These extremes highlight the importance of transparent counselling and shared decision-making, particularly because target titres and definitions of rebound were not captured and likely influence treatment intensity (7–9). Desensitisation was primarily halted for refractory titres or serious complications, yet 67% of centres indicated a willingness to reattempt after an interval of mostly 3–6 months, often combined with protocol intensification. Although robust peer−reviewed clinical evidence is currently lacking on the use of obinutuzumab in ABOi-KT, one centre has reported using this anti-CD20 antibody as a rescue approach. These findings highlight a progressive shift towards the aggressive, personalised management of very high-titre ABOi candidates.

Contemporary meta-analyses show that ABOi-KT achieve nearly comparable graft survival rates to those of ABO-compatible transplants (1). The Karolinska group first demonstrated that a single 375 mg/m² dose of rituximab could safely replace splenectomy and reduce rates of early AMR below 10% (5). Subsequently, antigen-specific IA was introduced to selectively remove anti-A/B antibodies with minimal plasma-protein loss (3). International cohorts report excellent patient and graft survival even at elevated isoagglutinin titres. including series specifically addressing very-high-titre recipients (7). Case reports describe successful transplants with initial isoagglutinin titres >1:8196 (15). However, Korean data show significant more antibody rebound and rejection when baseline IgG is ≥1:512 versus ≤1:256 (7). Nevertheless, many centres outside Germany have traditionally maintained titre cutoffs (often ≤1:256) to limit risk and costs, particularly where paired-donation programmes have offered an alternative strategy (16). In contrast, our survey suggests that German centres are at the forefront of advancing ABOi-KT to extreme isoagglutinin titres, reflecting their confidence in modern protocols and the availability of resources.

The absence of a universal isoagglutinin titre ceiling at almost half of the centres suggests a clinical paradigm in which any antibody level can be reduced with sufficient IA/PLEX and immunosuppression. This aggressive approach recognises the survival benefits of transplantation over dialysis for high-titre ABOi recipients (9, 13). However, studies conflict on bleeding risk. For example, one cohort study found higher rates of major haemorrhage in ABO-iKT patients (15% vs. 2%; p < 0.0005) and increased transfusion requirements (29% vs. 12%) (17). However, other studies have reported no significant difference compared to ABO-compatible transplants (18). Both PLEX and IA can induce thrombocytopenia, as well as the depletion of fibrinogen and clotting factors. Notably, bleeding occurs significantly more often after PLEX than after IA, which may explain the difference in complication rates (19). Point-of-care ROTEM can be used to guide coagulation therapy and reduce the risk of bleeding (20).

Decisions to terminate treatment are critical junctures; most centres cited either failure to reach a safe titre or the development of serious complications as triggers. The finding that 67% would retry underscores a prevailing optimism, but it also raises questions about futility thresholds: how many sessions constitute further therapy being non-beneficial? In light of the very high maximum treatment numbers reported (up to 36 sessions), the question of when continued escalation becomes futile is not merely theoretical, but directly impacts patient counselling, safety, and resource allocation. Until survival data linked to extreme isoagglutinin titres are available, these decisions will be based on individual clinical judgement rather than evidence-based guidelines.

A major barrier to harmonisation is the lack of standardised antibody-titre assays. Tube, gel-card and flow cytometry methods produce non-comparable dilution values, which makes it impossible to generalise isoagglutinin titre-based cutoffs across centres (21). To mitigate these discrepancies, adopting external, quality-assured, semi-automated column agglutination techniques might be useful. These techniques have been shown to be more sensitive and to produce less variation between laboratories than manual tube testing (21, 22). Similarly, there are no uniform criteria for defining desensitisation success or futility. Our results show that abortion decisions vary widely — some centres use informal session caps, while others rely on clinical judgement — leading to potential inequities in patient access. Standard operating procedures (SOP) for isoagglutinin titre measurement and consensus futility criteria (e.g. ‘abort if isoagglutinin titre remains >1:32 after “X” sessions’) would facilitate both patient counselling and multicentre outcome comparisons. Although these SOPs would be helpful, our survey shows that the number of extracorporeal treatments will remain an individual decision, given that the resilience of patients varies. Beyond assay variability, cost and logistical issues prevent uniform practice. Antigen-specific IA columns are expensive, single-use devices that are reimbursed variably across hospitals. They also do not work for induction in patients with dual ABO and HLA-incompatibility, a topic we did not address in this survey. Smaller centres may lack on-site IA capabilities and therefore use PLEX or limit the number of sessions. In terms of costs, the use of non-specific columns or the off-label reuse of specific columns may be a pragmatic solution (23). A U.S. economic analysis demonstrated that hospitalisation costs for ABOi-KT are roughly double those for ABO-compatible transplants (16). Furthermore, coordinating daily or alternate-day apheresis requires considerable staffing and scheduling resources. Ultimately, it is these real-world constraints rather than a lack of clinical evidence, that drive local desensitisation policies.

## Strengths and limitations

The strengths of our study include a high national response rate of 75% and a focus on extreme-titre cases, which are underrepresented in registries. Furthermore, our study is valuable because there is little information in the literature on how to deal with therapy-resistant titres. Collecting programme-level practices provides a valuable insight into the management of these cases in the real world and into variations between centres. Also, the level of detail reported is usually not present in register data. Ideally, surveys like this could be linked to register data and used to fill up gaps in information. However, this survey relies on self-reported centre-level data, which may introduce recall bias. While completion rates of the items were not perfect, we had a good response rate even in the open-ended questions. Plausibility of the given answers was good. For example, the number of reported ABOi-transplants was 411. Between 2020 and 2024, 2701 living donor kidney transplants were performed in Germany (DSO data). Of those, approximately 25% (≈675) would have been ABO-I KT according to registry estimates. Thus, the 411 reported cases correspond to about 60.9% of the expected national total, while the responding centres represent 64% of all German transplant centres (23/36).

We did not collect patient-level outcomes (e.g. rejection rates and graft survival) or detailed information on titre assay methods. This limits comparability across centres. With a 25% non-response rate, our results may overrepresent programmes that are more active. We also did not capture information on adjunctive therapies or the patient perspective on prolonged desensitisation. Furthermore, we recommend to study outcomes of patients with high titres e.g., in a national register. Finally, our results provide a snapshot of the situation at a given point in time, rather than indicating current or future trends.

## Future directions and research agenda

Based on the heterogeneity observed in this survey, several priorities for future research and clinical development have emerged. First, establishing a national or multinational registry that focuses on high-titre ABO-incompatible kidney transplantation would allow for the systematic linking of desensitization strategies with patient-level outcomes. Second, there is a clear need to standardize isoagglutinin titre measurement, including harmonized assay protocols and external quality assurance, to improve comparability across centres. Third, developing consensus-based futility criteria that integrate titre kinetics, clinical complications, and patient burden would support more transparent and reproducible decision-making regarding treatment discontinuation and reattempts. Fourth, given the substantial procedural intensity observed in some cases, future studies should incorporate structured analyses of resource utilization and cost-effectiveness, ideally in relation to dialysis avoidance and long-term outcomes. Finally, prospective studies that link protocol-level variables (e.g., apheresis intensity and rituximab exposure) with immunological and clinical endpoints are necessary to transition from experience-based to evidence-informed management of this high-risk subgroup.

## Data Availability

The original contributions presented in the study are included in the article/**Supplementary Material**. Further inquiries can be directed to the corresponding author.

## Supplementary material

Supplementary Table 1 ABOi questionnaire (translated).
