# Supplementary material for: ABO-incompatible kidney transplantation with high isoagglutinin titres: practice pattern and problem solving: a German multi-centre survey
Source: Front Immunol. 2026 Jun 5;17:1831754. doi: 10.3389/fimmu.2026.1831754 (PMC13281064; doi:10.3389/fimmu.2026.1831754)
Supplement: Supplementary Table 1 — ABOi questionnaire (translated). [file Table1.docx]

**Details on survey questions:**

1. **What is the maximum haemagglutinin titre at which your centre would perform an ABO-incompatible living kidney donation?**

- No upper limit: 48.1% (13/27)
- ≥ 1:2048: 11.1% (3/27)
- 1:1024: 25.9% (7/27)
- 1:512: 14.8% (4/27)

1. **Which hemagglutinin titres do you use? (Multiple selections possible)**

Combinations:

- IgM and IgG: 62.9% (17/27)
- Only IgG: 33.3% (9/27)
- Only IgM: 3.7% (1/27)

Totals for each titre:

- IgG: 96.3% (26/27)
- IgM: 66.7% (18/27)

1. **Which test method do you use to determine immunoglobulin titres?**

- Gel cards: 55.5% (15/27)
- Tube test: 33.3% (9/27)
- Other: 11.1% (3/27): unknown (2); both (1)

1. **Is there a specific protocol/pre-treatment for ABO-incompatible living kidney donation with high isoagglutinin titres? (Multiple selections possible)**

- Standard dose rituximab (375 mg/m2 BSA): 76.9% (20/26)
- Increased dose: 0%
- Additional dose:11.5% (3/26)
- Other: 11.5% (3/26)
  - 500mg rituximab (instead of normal dose) (1)
  - additional 75mg thymoglobulin (1)
  - additional dose of rituximab if titres do not decline sufficiently (1)

1. **When do you start oral immunosuppression?**

- 1 week prior to kidney transplant: 36% (9/25)
- 2 weeks prior to kidney transplant: 16% (4/25)
- 4 weeks prior to kidney transplant: 8% (2/25)
- Other (please define): 40%:
  - 5 weeks before planned kidney transplant (1)
  - with rituximab (ca 4 weeks before planned transplantation) (2)
  - 3 weeks before extracorporeal treatment (1)
  - 2 weeks before extracorporeal treatment (1)
  - 1 week before extracorporeal treatment (1)
  - 1 day before extracorporeal treatment (1)
  - at hospital admission (1)
  - 3-4 days before planned transplantation (1)
  - depending on isoagglutinin-titre (1)

1. **When do you administer rituximab?**

- 1 week prior to kidney transplant: 4% (1/25)
- 2 weeks prior to kidney transplant: 0%
- 4 weeks prior to kidney transplant: 64% (16/25)
- Other (please define): 32 % (8/25)
  - 1 day prior to kidney transplant (1)
  - 1-2 weeks prior to extracorporeal treatment (1)
  - depending on titre, only if >=1/16: 2 weeks prior to extracorporeal treatment (1)
  - 3 weeks prior to extracorporeal treatment (2)
  - 4 weeks prior to extracorporeal treatment (2)
  - 5 weeks prior to kidney transplant (1)

1. **Do you administer immunoglobulins?**

- Yes, as with low titre: 24% (6/25)
- Yes, only with high titre: 8% (2/25)
- No: 68% (17/25)

1. **Which form of extracorporeal treatment does your center *prefer* for patients with high isoagglutinin titres? (In case of combination therapy, multiple answers possible)**

Combinations:

- only ABO-specific adsorbers: 38% (10/26)
- AB0-specific and/or unspecific adsorbers: 4% (1/26)
- AB0-specific adsorber and/or PLEX: 35% (9/26)
- AB0-specific and/or unspecific adsorbers and/or PLEX: 12% (3/16)
- only AB0-unspecific: 4% (1/26)
- AB0-unspecific and/or PLEX: 4% (1/26)
- only plasmapheresis (PLEX): 4% (1/26)

Totals for specific methods used in preferred treatment:

- Immunoadsorption with ABO-specific adsorbers: 88.46% (23/26)
- Immunoadsorption with unspecific adsorbers: 23.1% (6/26)
- PLEX: 53.8% (14/26)

1. **What exchange volume do you use for immunoadsorption (IA) with ABO-specific columns? (Please provide only one answer)**

No answer: 22% (6/27), no valid/quantitative answer: 15% (4/27)

- - What multiple of the plasma volume do you use (as in low titres)?
    - median 3 (IQR 2.5-3; Range 1-5)
    - in two instances absolute volume given: 12 l and 14 l
  - What multiple of the plasma volume do you use (increased in high titres)
    - 7/14 (50%) of centres (that use method and gave quantitative answer) increase plasma volume in high titres
    - Median 4 (IQR 3-4; Range 1.5-6)
  - Method not used: 3

1. **What exchange volume do you use for immunoadsorption (IA) with ABO non-specific columns? (Please provide only one answer)**

No answer: 33% (9/27), no valid/quantitative answer 4% (1/27)

- - What multiple of the plasma volume do you use (as in low titres)?
    - median 2,5 (IQR 2.3-4; Range 1.5-5)
    - in one instance absolute volume given: 12 l
  - What multiple of the plasma volume do you use (increased in high titres)
    - Median 3 (IQR 2.75-3.75; Range 2-6)
    - 4/8 (50%) of centres (that use method and gave quantitative answer) increase plasma volume in high titres
  - Method not used: 9

1. **What exchange volume do you use for plasma exchange (PLEX)? (Please provide only one answer)**

No answer 22% (6/27), no valid/quantitative answer 7% (2/27)

- - What multiple of the plasma volume do you use (as in low titres)?
    - median 1,4 (IQR 1-2.1; Range 1-3)
    - in four instances absolute volume given (2.5l; 2-3l; 2-2.5l; up to 4l)
  - What multiple of the plasma volume do you use (increased in high titres)
    - Median 2 (IQR 2-2.5; Range 2-3)
    - 3/16 (19%) of centres (that use method and gave quantitative answer) increase plasma volume in high titres
  - Method not used: 3

1. **What is the maximum number of extracorporeal treatments that have been performed at your centre to reduce isoagglutinin titres prior to ABO-incompatible kidney transplantation?**
   - with successful transplantation (24 answers):
     - Median: 16.5, IQR: 10-22.5; Range: 5-36
     - 2 transplants after more than 25 treatments
   - without kidney transplantation (12 valid/plausible answers):
     - Median: 16, IQR: 12-20.5; Range: 7-30
     - some centers misunderstood the question and indicated number of patients with discontinuation of treatment
2. **What measures do you take at your center if the isoagglutinin titres does not decrease as expected? (Multiple selections possible)**

No answer 1/27

- - Additional administration of immunoglobulins: 12%
  - Additional dose of rituximab: 38%
  - Higher exchange volume: 69%
  - Switch from PLEX to immunoadsorption: 15%
  - Switch from immunoadsorption to PLEX: 77%
  - Additional extracorporeal treatment: 73%
  - Other (free text): 31%:
  - Switch from unspecific to specific adsorption (1/26)
  - Switching brand of specific adsorber (1/26)
  - Additional steroid bolus (500 mg) (1/26)
  - Intermittent PLEX (1/26)
  - Prolonged immunoadsorption (1/26)
  - discontinue treatment (1/26)
  - *did not have that situation* (1/26)
  - additional rituximab plus PLEX (1/26)

1. **When do you discontinue (abort) the treatment? (multiple answers plus free text)**

No answer 3/27

- - Specific number of treatments:
  - 7 (1), 10 (1), 12 (2)
  - about 20, if target titre does not seem attainable (1)
  - Individual number of treatments, 10 up to now (1)
  - At repeated titre rebound above center-specific threshold:
  - Yes: 16 centers
  - No: 1 center
  - Situation never occurred: 2 centers
  - Unclear answer: 1 center
  - With complications (please state complication):
  - Infections (4)
  - thrombocytopenia (1)
  - allergic reaction (3)
  - hypocalcemia (1)
  - clinical condition of the patient (2)
  - Other (please describe):
  - Posterior reversible encephalopathy syndrome (PRES) (1)
  - on request of the patient (3)
  - discontinuation never occurred (2)
  - medical problems (1)

1. **Would you make a second attempt at titre reduction?**

No answer: 3/27

- Yes: 16/24 (67%)
- No: 8/24 (33%)

2 “no” answers were adjudicated as a yes, as the respective centres stated in question 16, that they would attempt a second try under the condition that titres declined (1), or discontinuation of treatment was due to adverse events of treatment (i.e. infections) (1)

1. **Under what conditions would your centre make a second attempt at titre reduction? Please describe briefly. (Open ended response, multiple answers possible)**

No answer: 1/16 centres that would make a second attempt, valid answers: 15

- Request of patient (5)
- Patient in good condition (4)
- discontinuation of treatment was due to adverse events of treatment (i.e. infections)/not due to refractory isoaggluttinine-titre (3)
- Dependent on isoagglutinin titre (3)
- generally offered (2)
- after antibody-depleting therapy (i.e. second dose of rituximab) if applicable (2)
- dependent on cause for discontinuation (2)
- After resolution of complications (2)
- treatment well tolerated, short waiting time/preemptive transplant possible (1)
- change of extracorporeal treatment (1)
- after 6 months (1)

1. **After what time interval would you attempt a second round of titre reduction at your centre? If “Other” please elaborate.**

Valid answers: 15:

- After 3 months: (3/15) 20%
- After 6 months: (7/15) 47%
- After 12 months: (0) 0%
- Other: (5/15) 33%
  - 4 weeks (after second dose of rituximab at previous discharge) (1)
  - 3-6 months (1)
  - individual basis (1)
  - after resolution of complication that led to discontinuation (2)

4 answers in the “Other” category were not counted, as they were given by centres that do no second attempts. 1 centre that would make a second attempt did not provide an answer.

1. **In case of a second attempt: Do you take additional measures? If yes, which ones?**

Valid answers: 16

- No 50% (8)
- Yes 50% (8)
- If yes, with measures (multiple answers possible):
  - additional dose of rituximab (6)
  - higher exchange volume (2)
  - change of column (1)
  - obinutuzumab (1)

1. **How many ABOi transplantations have been performed at your centre in the last 5 years?**

Valid answers: 23

Mean: Median: 16; IQR: 8-23.5; Range: 3-50, Total: 411 ABOi-kidney transplants
